# P-885. Eight Years In, Still Going Strong: A Single-Center ASP’s Sustained Success in Optimizing and Reducing Antibiotic Use

**DOI:** 10.1093/ofid/ofaf695.1093

**Published:** 2026-01-11

**Authors:** Ivan Gutierrez, Juan Londono, Alexandra Jimenez, Adriana Cuenca, Guillermo Eljadue, Naddya Bermudez

**Affiliations:** Clínica Infantil Santa María del Lago, Clínica Infantil Colsubsidio, Bogota, Distrito Capital de Bogota, Colombia; Clínica Infantil Colsubsidio, Bogotá, Distrito Capital de Bogota, Colombia; Clínica Infantil Colsubsidio, Bogotá, Distrito Capital de Bogota, Colombia; Clinica Infantil Colsubsidio, Bogota, Distrito Capital de Bogota, Colombia; Clinica Infantil Colsubsidio, Bogota, Distrito Capital de Bogota, Colombia; Clinica Infantil Colsubsidio, Bogota, Distrito Capital de Bogota, Colombia

## Abstract

**Background:**

Antibiotic stewardship programs (ASP) effectively optimize antimicrobial use in pediatric settings. In late 2016, our 180-bed pediatric hospital in Bogotá, Colombia, launched an ASP anchored in prospective audit and feedback, supported by expanded technology for real-time interventions. Over time, the program addressed peritonitis, Mycoplasma pneumoniae, and oncology-specific needs. We aimed to evaluate its long-term impact on hospital-wide antibiotic consumption.

**Figure:**
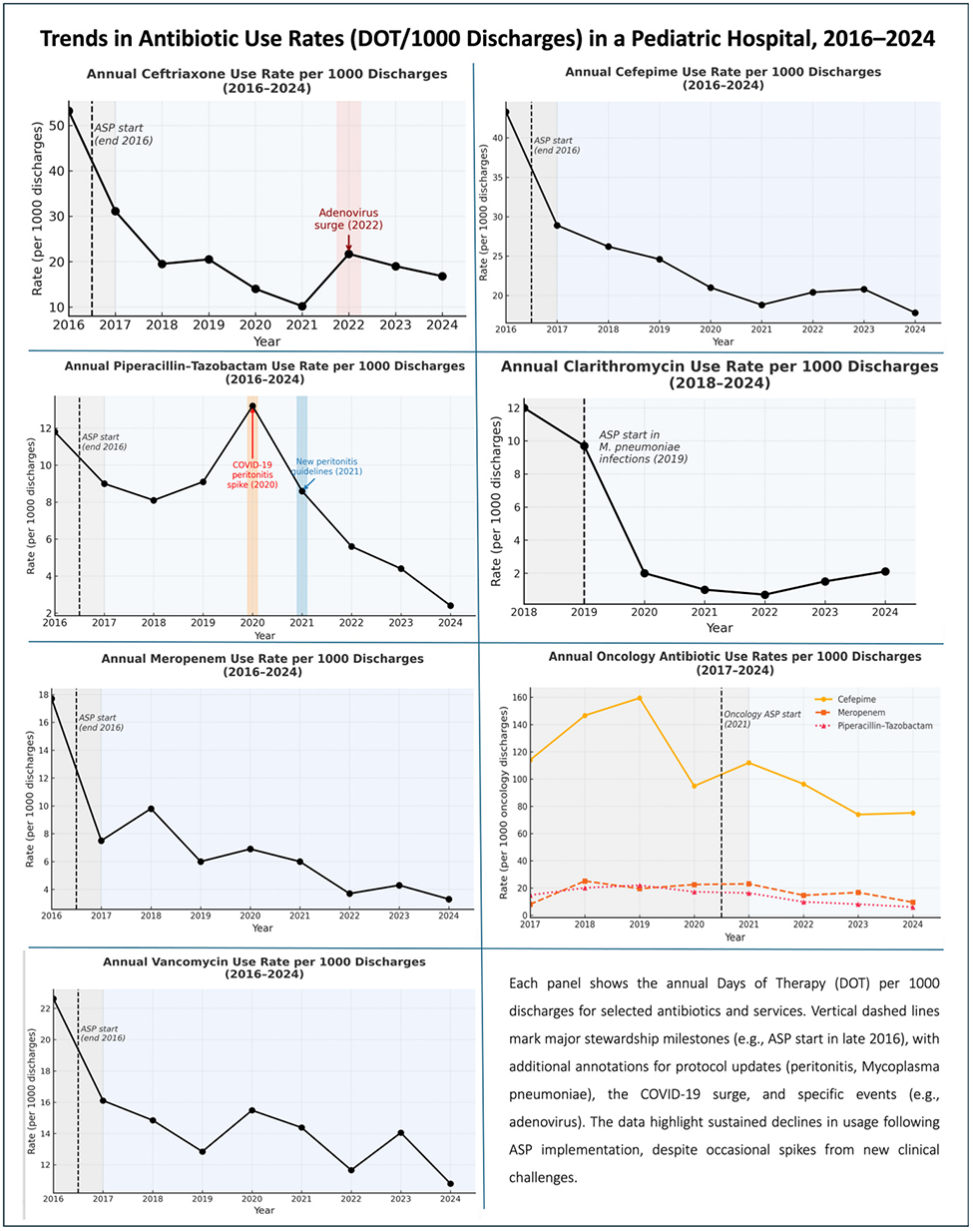
Trends in Antibiotic Use Rates (DOT/1000 Discharges) in a Pediatric Hospital, 2016–2024 Each panel shows the annual Days of Therapy (DOT) per 1000 discharges for selected antibiotics and services. Vertical dashed lines mark major stewardship milestones (e.g., ASP start in late 2016), with additional annotations for protocol updates (peritonitis, Mycoplasma pneumoniae), the COVID-19 surge, and specific events (e.g., adenovirus). The data highlight sustained declines in usage following ASP implementation, despite occasional spikes from new clinical challenges.

**Methods:**

We conducted a retrospective study (January 2016–December 2024). Antibiotic use was measured as days of therapy (DOT) per 1000 discharges in five services: Emergency Department, Pediatric Intensive Care Unit, Intermediate Care, General Pediatric Wards, and Oncology. Monthly DOT were summed annually, and total discharges served as the denominator. We assessed trends in broad-spectrum and targeted agents (cefepime, meropenem, vancomycin, piperacillin-tazobactam, clarithromycin) versus a 2016 baseline

**Results:**

Overall, annual antibiotic consumption (DOT per 1000 discharges) decreased substantially following ASP implementation. Cefepime fell from 38 in 2016 to 11 in 2024 (a 71% reduction). Meropenem dropped from 16 to 7 (56% reduction), and Vancomycin from 22 to 9 (59% reduction) over the same interval. Clarithromycin VO consumption declined from 12 at its 2019 peak to 2 in 2024 (an 83% decrease). In Oncology, stewardship efforts coincided with a reduction in cefepime use from 130 to 78 DOT/1000 discharges (40% decrease). Notably, transient spikes occurred during the COVID-19 pandemic (2020) and after updated peritonitis guidelines, yet the long-term trend remained downward across most agents.

**Conclusion:**

An ASP combining audit-and-feedback with technology-driven interventions achieved substantial, sustained antibiotic reductions across multiple services. Despite occasional upticks, usage remained well below pre-ASP levels, demonstrating the resilience of a proactive stewardship model. Ongoing refinements are critical to maintaining these gains and mitigating antimicrobial resistance in pediatric care.

**Disclosures:**

All Authors: No reported disclosures

